# Methods of Preparation and Performance Evaluation of ABS/Mineral Microsphere Composites Produced through FDM and Compression Molding

**DOI:** 10.3390/ma15145021

**Published:** 2022-07-19

**Authors:** Panagiotis M. Angelopoulos, Nikolaos Rafael Vrithias, Zacharias Viskadourakis, Petros Tsakiridis, Konstantinos C. Vasilopoulos, Antonis Peppas, Georgios Asimakopoulos, Anastasia V. Spyrou, Michael A. Karakassides, Maria Taxiarchou, George Kenanakis

**Affiliations:** 1 School of Mining and Metallurgical Engineering, National Technical University of Athens, Zografou Campus, 9 Iroon Polytechniou Str., Zografou, GR-157 80 Athens, Greece; ptsakiri@central.ntua.gr (P.T.); peppas@metal.ntua.gr (A.P.); taxiarh@metal.ntua.gr (M.T.); 2 Institute of Electronic Structure and Laser, Foundation for Research and Technology-Hellas, N. Plastira 100, Vasilika Vouton, GR-700 13 Heraklion, Greece; nvrithias@materials.uoc.gr (N.R.V.); zach@iesl.forth.gr (Z.V.); gkenanak@iesl.forth.gr (G.K.); 3 Department of Materials Science and Technology, University of Crete, GR-700 13 Heraklion, Greece; 4 Department of Materials Science and Engineering, University of Ioannina, GR-451 10 Ioannina, Greece; kovasil@auth.gr (K.C.V.); asimakopoulos.geo@gmail.com (G.A.); anast.spirou@yahoo.gr (A.V.S.); mkarakas@uoi.gr (M.A.K.)

**Keywords:** perlite microspheres, composites, fused deposition modeling, flammability, hot-pressing, compression molding, 3D printing, composite filaments

## Abstract

The use of amorphous microspheres as filler in composites is promising due to their light weight, low cost, incombustibility, and the ability to alter relevant properties of the final composite. Contrary to glass spheres, perlite microspheres are much cheaper and can be tailor-made to facilitate purpose-oriented alteration of the final composite. We report the use of perlite microspheres for the preparation of: (1) composites, through a compression molding (hot pressing) technique; and (2) composite filaments, in a single screw extruder, as well as their use for sample printing through Fused Deposition Modeling (FDM). Proper characterization of the produced composites allows for their evaluation in terms of physical, thermal, and mechanical properties and with regards to the manufacturing technique, the filler fraction, and size. Composite samples of acceptable quality in terms of filler survival and dispersion as well as mechanical properties were produced through compression molding using fine expanded perlite microspheres (<90 μm) up to an infill ratio of 40 vol.%. Fine fillers (<90 μm) performed well in FDM, allowing printing of composite dogbone samples with a higher Young’s modulus and elongation and similar ultimate tensile strength compared to benchmark, up to an infill ratio of 20 vol.%. Composite samples present a slightly lower burning rate compared to those produced solely by ABS. Perlite microspheres present good workability in both applications, possessing satisfactory performance as filler in the composites, and can thus be assumed a promising multifunctional filler for various thermoplastics considering their low price, environmental impact, and fire rating.

## 1. Introduction

Expanded perlite is a widely used material that possesses attractive properties rendering it a promising filler for composites. It is lightweight, spherical, inorganic, flame-retardant, and low cost. Furthermore, it exhibits considerable thermal and sound insulating properties as a consequence of its foamy structure [1]. Such properties have attracted the interest of researchers, who have investigated its effect on most important properties of composites prepared using common organics as binder. Akin-Öktem et al. investigated the mechanical properties of perlite-filled gamma-irradiated polypropylene (PP), using perlite of 0.9 g/cm^3^ density. They noted an improvement in ultimate strength and tensile strength along with a decrease in elongation of irradiated perlite-filled PP samples after irradiation [2]. Raji et al. studied the effect of the addition of expanded and unexpanded perlite on the solid and melt state of PP composite produced by twin-screw extrusion combined with compression molding [3]. The researchers claimed that both the higher mechanical and rheological properties of raw perlite composites can be attributed to the presence of hydroxyl groups on their surface, and noted that raw perlite might improve the thermal resistance and the fire retardancy of polymer composites as well as their mechanical properties. Atagür et al. studied the mechanical, thermal, and viscoelastic properties of expanded perlite–high-density polyethylene composites (HDPE) prepared in a thermokinetic mixer and subsequently hot pressed at 180 °C [4]. They observed a reduction in thermal conductivity by 7.9% for 20 wt.% incorporation of perlite particles along with a better increment of the Young’s modulus and flexural strength. A perlite–HDPE system was studied by Lapčík et al. as well, who noted a strong effect of perlite on the mechanical and thermal properties of the composite [5]; an increase in the Young’s modulus and decrease in the elongation at break was observed with increasing filler concentration. In that study, the composite was formed through injection molding, while the density of submicron perlite was 1.1 g/cm^3^. Gerhardt de Oliveira et al. prepared polystyrene (PS)-expanded perlite composites using twin screw extrusion and evaluated their thermal, mechanical, and rheological properties, noting a reduction in tensile strength due to the presence of destroyed filler particles acting as tension concentrators [6]. An increase in viscous behavior was observed due to the fillers’ lubricant effect on PS chains. In another study, ethylene–propylene–diene rubber modified with phenolic pulp was mixed with expanded perlite. Composite samples were prepared in a single screw extruder and vulcanized at 180 °C in a hot press at a pressure of 50 bar, however, the perlite particles did not survive the manufacturing process [7]. Alsaadi and Erkliğ investigated the effect of fine expanded perlite (diameter of 1–35 μm and bulk density of 0.16 gr/cm^3^) on interlaminar fracture and mechanical behavior of glass fabric/epoxy composites [8]. The researchers noted that the addition of perlite caused significant improvement in interlaminar fracture, tensile and flexural strength, flexural modulus, and failure strain.

Another dynamically growing field where polymer composites find application is 3D printing. Fused Deposition Modeling (FDM) constitutes the most widespread 3D printing technique, in which a composite is formed in filaments using a single or double screw extruder with a mixture of polymer pellets and filler. The purpose of incorporating the filler is to produce a composite which can exhibit tuned properties that are not present in the parent materials, commonly focusing on rheology; by this printing quality, it is possible to achieve various mechanical and physical properties, different appearance and texture, and even to render materials conductive or susceptible to magnetism. For this purpose, various types of fillers have been used, including ceramics, C-based materials, minerals, and metallic, glassy, and wood-based materials [9]. The use of expanded perlite as filler has been limited. Spoerks et al. prepared a composite filament consisting of expanded perlite and polypropylene that was used in FDM [10]. They noted that the use of fine expanded perlite (diameter below 20 μm) resulted in a significant reduction in shrinkage and warpage. The addition of amorphous polyolefin to the composite resulted in an improved Young’s modulus and a slight reduction in yield stress. The expansion level of the filler was low, with a density of 1.7 g/cm^3^, while the density of the raw material was 2.2 g/cm^3^. In our previous preliminary study, we investigated the mechanical properties of dogbone samples printed using composite filaments produced with ABS and expanded perlite in different proportions [11]. The granulometry of the filler ranged from a few microns to 500 μm, with the bulk density ranging from 58 to 168 kg/m^3^ for different granulometric fractions. It was shown that fine fillers (<90 μm) tend to survive the extrusion process, while infill ratio should not exceed 20 vol.%.

In the current study, we present a more systematic investigation of the use of expanded perlite microspheres as filler in ABS composites. These are formed through two common compounding techniques, namely, compression molding and screw extrusion. Three grades of fine expanded perlite microspheres of different density and strength, were prepared in a Vertical Electric Furnace and used as filler for the preparation of composites with infill ratios of up to 40 vol.% for compression molding samples and 20 vol.% for injection molding samples. Filaments produced by injection molding were fed into a commercial FDM printer and dogbone samples were printed. Benchmark and composite samples were subjected to characterization of their crucial mechanical, physical, and thermal properties as well as morphological observation through SEM with the aim of evaluating their properties and identifying the effects of the filler characteristics and infill ratio on their quality.

## 2. Materials and Methods

### 2.1. Filler Production and Characterization

Hydrous rhyolitic (volcanic) glass was used for the preparation of expanded microspheres. Mechanical sieving was applied to secure production of two raw material grades with size distributions below 150 and 400 μm. Subsequently, the raw materials were fed continuously into the VEF at a feed rate of 5 kg/h. Air was injected at the top of the furnace and concurrently to the particle flow at a rate of 30 L/min, facilitating particle dispersion and flow homogeneity in the furnace heating chamber. More information regarding the experimental setup, production procedure, and major physical properties of the expanded microspheres can be found elsewhere [12,13,14]. The furnace temperature was set according to the desirable expansion ratio. Two grades of expanded microspheres with different bulk densities were produced with a fine feed (<150 μm) through the application of a specific temperature profile. The coarser grade (<400 μm) was subjected to expansion and subsequent sieving for the production of expanded microspheres with different granulometries (<90 μm, 90–200 μm, and 200–350 μm). The bulk density of the expanded microspheres was determined by measuring the weight of the sample in a volumetric cylinder and dividing it by the volume occupied. Furthermore, the real or skeletal density was determined in a Quantachrome Helium micropycnometer (Anton Paar, Graz, Austria). The particles morphology and microstructure were both evaluated by scanning electron microscopy (SEM) using a Jeol 6380 LV microscope (Jeol, Tokyo, Japan); the experimental conditions involved 15 and 20 KV accelerating voltage using a backscattered electron detector. The crushing resistance of the fillers was determined through a standardized procedure by measuring the force required to compress the filler by 5 cm in a universal testing machine [15]

### 2.2. Compression Molding

The two different grades of filler were mixed with Acrylonitrile-Butadiene-Styrene (ABS). Terluran Hi-10 grade ABS granules were supplied by INEOS (Styrolution, Frankfurt, Germany) in different *v/v*% contents (0%, 5%, 10%, 20%, and 40% *v/v*). ABS was chosen as a polymeric material due to its low density, high impact resistance, and mechanical hardness. ABS is used in many consumer products, and is resistant to acids, alkalis, alcohols, and mineral oils, to name only a few [16].

A selected amount of ABS pellets and microspheres were added to the desirable volume fraction, mixed in a mechanical homogenizer, and the mixture was subjected to overnight drying at 60 °C using a typical laboratory oven (Memmert UNP 500, Memmert GmbH + Co. KG, Schwabach, Germany). Expanded ABS/perlite mixtures were placed in homemade copper molds in a preheated oven at 140 °C for 5 h in order to eliminate humidity and melt the polymeric matrix, and afterwards the molds were forwarded to a hydraulic press equipped with dual element heating plates (Color-King SGS, Fuzhou, China) preheated at 140 °C for 20 min. Finally, pressure of 4MPa was applied at 170 °C for 40 min in order to produce ABS/perlite cylinders with a diameter of 20 mm and height of 16 mm.

The thermal behavior of the obtained composites and the reference ABS material was studied using a DSC214 Polyma Differential Scanning Calorimeter (NETZSCH manufacturer, Selb, Germany). Samples between 5–20 mg were tested in a dynamic heating/cooling mode in the temperature range from −150 °C to 200 °C at a heating rate of 10 °K/min under a nitrogen atmosphere. Controlled compression trials were applied on an Instron 5500 (USA) Universal Testing Machine (UTM) with crosshead speed of 1 mm/min to evaluate the mechanical strength of the composites under compressing conditions.

Pure ABS and expanded ABS/perlite mixtures were subjected to measurement of thermal conductivity and effusivity using a TCi thermal conductivity analyzer (C-Therm Technologies Ltd., Fredericton, NB, Canada) using the modified transient plane method. Five different pieces of each concentration were measured for the calculation of mean and relative standard deviation values of thermal conductivity (k) and effusivity (e).

### 2.3. Composite Filament Preparation and FDM

**Figure 1:**
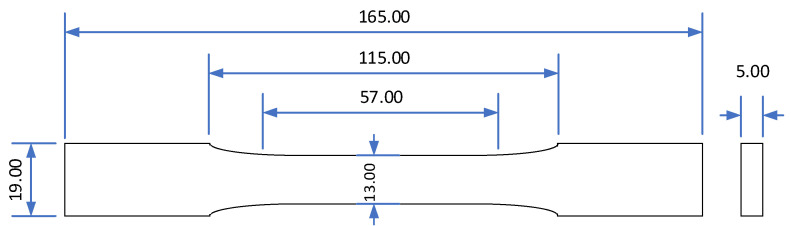
Dimensions of dogbone samples in accordance with ASTM D638-14 [17]. All dimensions in mm.

The incorporation of microspheres in ABS matrix and the formation of composite filaments was carried out in a single screw Noztek Pro extruder (Noztek, Shoreham-by-Sea, UK). A selected amount of ABS pellets and microspheres were added to the desirable volume fraction and mixed in a mechanical homogenizer, then the mixture was subjected to overnight drying at 60 °C. Among different extrusion temperatures applied in the range between 200 and 250 °C, optimal manufacturing conditions were achieved at 230 °C in terms of filament production stability and macroscopic structural consistency. A proper nozzle was installed to allow production of filaments with diameter of 1.75 ± 0.05 mm. Neat ABS and composite filaments were used to print dogbone samples on a MakerBot replicator 2X desktop 3D printer (Makerbot, New York, NY, USA). The following printing conditions were applied: 0.4 mm layer height, 100% solid infill, crosshead speed 20 mm/min, 0.8 mm nozzle diameter, nozzle temperature 235 °C, 130 °C bed temperature, and 45° deposition orientation. The dimensions of the dogbone samples are shown in Figure 1, and are in accordance with the ASTM D638-14 “Standard Test Method for Tensile Properties of Plastics” Type I [17]. Tensile strength was measured in an Instron 5500 (Shakopee, MI, USA) Universal Testing Machine (UTM) with a crosshead speed of 5 mm/min. For microstructure detection of composite filaments and FDM samples as well as compression molding samples, SEM was performed in polished sections produced by vacuum impregnation of the selected sample in a low viscosity epoxy resin. After removing a small surface by cutting with a micro-saw, the sample was subjected to grinding and polishing with 1μm diamond paste on a lapping disk.

Three printed dogbone samples were reshaped to a size of 125 mm length and 13 mm width and subsequently subjected to flammability testing according to the ASTM D 635-03 standard procedure “Standard Test Method for Rate of Burning and/or Extent and Time of Burning of Plastics in a Horizontal Position” to determine their burning rate [18].

## 3. Results

### 3.1. Microsphere Properties

The control of the feed size and the selection of different thermal processing conditions in the Vertical Electric Furnace allowed the production of perlite microspheres with different properties in terms of density, as seen in Table 1. Normally, an increase in the expansion temperature led to the production of samples of lower bulk density. Thus, by applying temperatures below 1000 °C, F1 and F2 grades were produced with bulk density of 375 and 469 kg/m^3^, respectively, while F3 grade was produced at 1030 °C, obtaining a bulk density of 198 kg/m^3^. For skeletal density, the considered volume of the sample is the grain volume, including the space occupied by inaccessible pores in the grains. On the contrary, for loose bulk density, open voids and interparticle space are not taken into consideration. Thus, skeletal density is critical for lightweight fillers because it allows estimation of the effect of the filler on the density of the composite. ABS density is estimated at 1–1.05 g/cm^3^, which is lower compared to that of the F1 and F2 fillers and higher compared to that of the F3 filler [19]. Crushing resistance ranges from 1.9 to 6.2 MPa, with a lower value identified for the most lightweight grade (bulk density of 0.198 g/cm^3^) and the highest for the heavier grade (bulk density of 0.469 g/cm^3^), as expected due to the higher porosity of the most lightweight grades [12,14].

**Figure 2:**
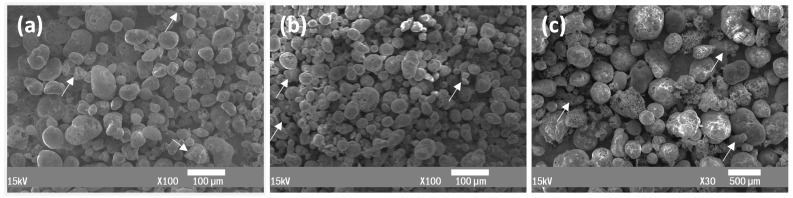
SEM images of three produced microsphere grades: F1 at 100× magnification (**a**); F2 at 100× magnification (**b**); and F3 at 30× magnification (**c**). The white arrows show non-expandable particles.

The morphology of all filler grades produced is presented in Figure 2. In microspheres F1 and F2 (Figure 2a,b), both samples mainly consist of rounded closed-surface particles with high sphericity and sizes up to 100 μm, while the F3 sample presents many grains with higher surface porosity. A small portion of non-expandable crystalline phases, such as biotite, quartz and plagioclase, are usually present in perlite deposits and are spread among all samples, appearing as tiny angular particles located on the sample base [12]. As more grain fragments are produced during the extended thermal treatment, more are present in microsphere grade F3, which was treated at 1030 °C, than in F1 and F2, which were produced at 990 and 930 °C, respectively.

### 3.2. Compression Molding Samples

In this section, we present the studied properties of the samples produced using neat ABS and various ABS–filler combinations through compression molding.

#### 3.2.1. Morphology and Density

**Figure 3:**
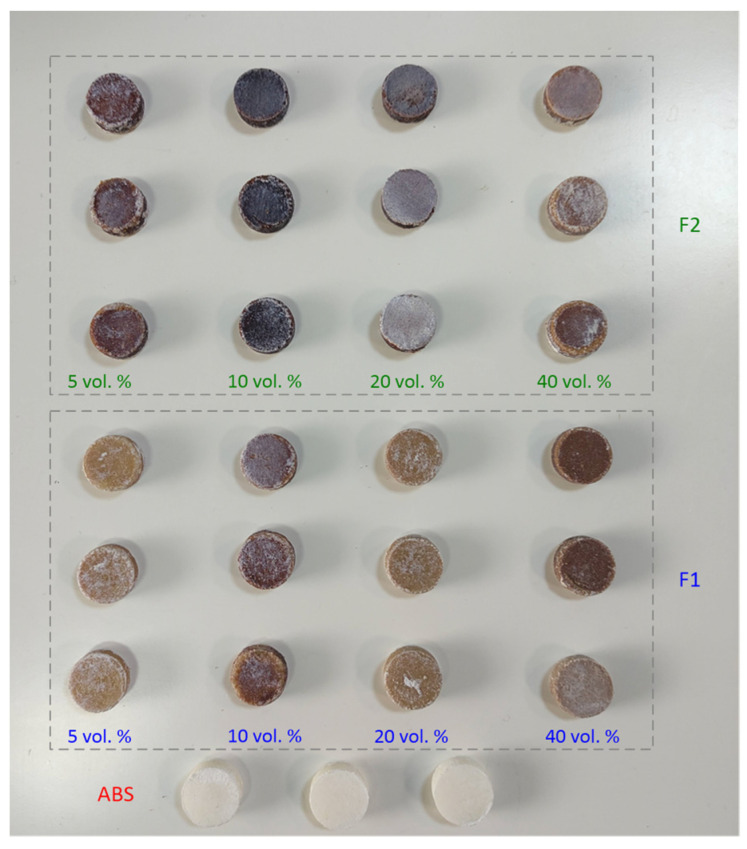
Samples produced by compression molding from neat ABS and F1 and F2 fillers in different proportions.

Figure 3 depicts all samples produced using neat ABS and ABS–microsphere composites. Cylindrical samples with 20 mm diameter and 16 mm height were produced using fillers F1 and F2 in different proportions in triplicate.

**Figure 4:**
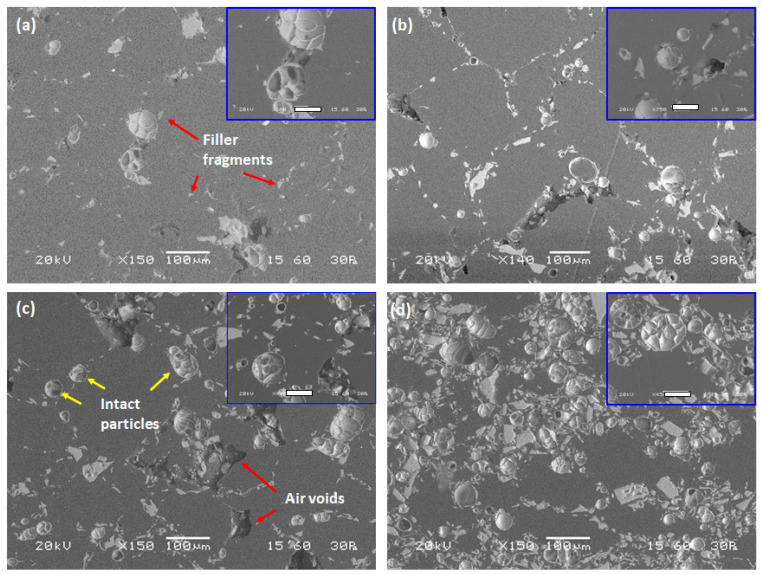
SEM images of cross-section of samples prepared by compression molding with different proportions of F1: (**a**) 5 vol.%, (**b**) 10 vol.%, (**c**) 20 vol.%, and (**d**) 40 vol.%. Regions of interest are depicted in higher magnification at the top right of each image with bar length equal to 40 μm.

The morphology at the cross-sections of samples produced using F1 filler in different proportions is presented in Figure 4.

The sample with minimum filler content (Figure 4a, 5 vol.%) presents few intact filler grains. With respect to the spherical particles, filler fragments dispersed along the composite matrix were identified; these may coexist in the filler or be produced during preparation of the composite sample through compression molding. Air voids can be observed, mainly in the vicinity of the filler, although dispersed along the ABS matrix as well. More intact filler particles are identified with an infill ratio of 10 vol.%, with a parallel increase in the presence of fragments (Figure 4b). Elongated air voids are present, which are randomly oriented. The presence of grain fragments in the air void might denote that the voids are attributed to the collapse of the filler, probably during compression molding. More air voids can be identified in the sample with a filler content of 20 vol.% (Figure 4c). Many intact particles of various sizes can be identified in the sample. Filler clusters are present without affecting the dispersion homogeneity on the macroscopic level. The size of the air voids is similar to those seen in the sample with 10 vol.% filler. The sample with maximum filler content (Figure 4d, 40 vol.%) presents a dense network of microspheres which are well dispersed along the ABS matrix. With respect to the spherical microspheres, compact particles of irregular shape were identified. Such particles constitute either inadequately expanded perlite particles or non-expandable particles with a high proportion of biotite, quartz, and/or plagioclase. It is worth noting that no air voids could be identified in this case.

As can be seen from the observation of the microstructure of the samples, the filler was adequately dispersed in the ABS matrix regardless of filler content. A significant amount of filler remained intact after compression molding, forming a dense and homogeneous network of multicellular microspheres with diameter below 100 μm, as seen in the SEM images (Figure 4). The interfacial adhesion of the filler to the ABS matrix is satisfactory as no air voids could be identified on the external surface, as revealed by the high magnification micrographs.

**Figure 5:**
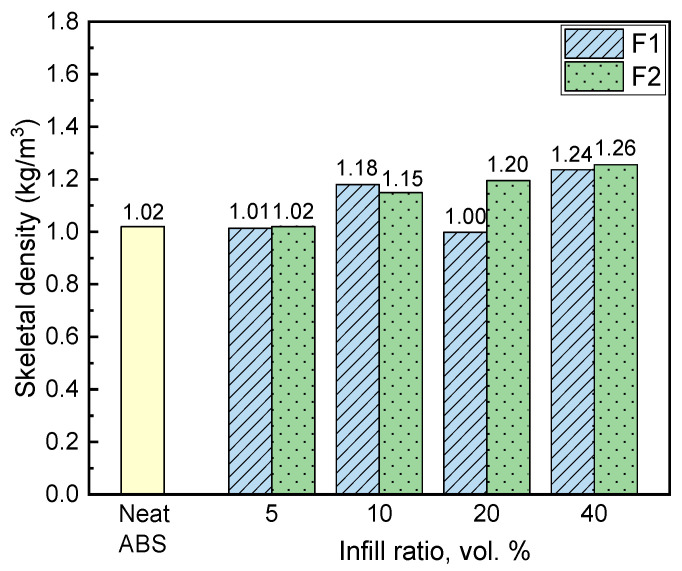
Skeletal density of neat ABS and composite samples.

Figure 5 presents the skeletal density of the samples prepared using neat ABS and fillers F1 and F2 in different proportions.

A trend towards increasing density with increase infill ratio is observed, as expected, as the density of both the F1 and F2 fillers is slightly higher compared to ABS (Figure 5). With the same infill ratio, composite skeletal density is lower when F1 filler is used, probably as a consequence of its lower skeletal density compared to F2 (375 and 469 kg/m^3^, respectively). The lower density of composites with 5 and 20 vol.% infill ratios of F1 filler compared to neat ABS revealed the existence of air voids in the samples, which is in line with the micrographs presented in Figure 4.

#### 3.2.2. Mechanical Properties of Compression Molding Samples

**Figure 6:**
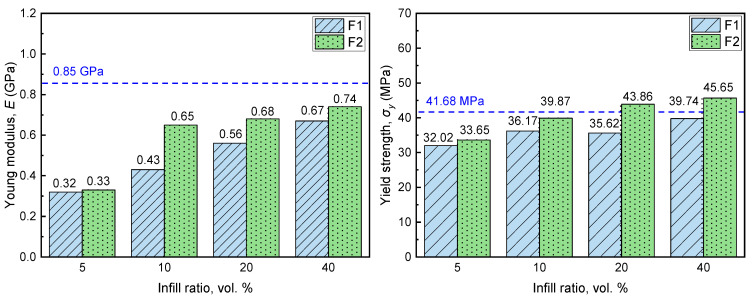
Young’s modulus and yield strength of samples prepared using different proportions of F1 and F2 fillers. Horizontal dashed blue lines denote respective property value for sample prepared solely with ABS.

The evaluation of the composites’ strength under compression was carried out by subjection of the samples to controlled compression and estimation of Young’s modulus and yield strength. The obtained values for the composite samples are presented in Figure 6 together with the respective values for the sample produced using neat ABS, presented as dashed blue lines.

The Young’s modulus of the produced sample is higher compared to that of composite ones, presenting a value of 0.85 GPa, while for composite samples the Young’s modulus increases with increasing infill ratio, obtaining maximum value of 0.67 and 0.74 GPa for an infill ratio of 40 vol.% for F1 and F2 filler grades, respectively [20]. A significant reduction in Young’s modulus is observed for the sample with minimum infill ratio (5 vol.%); compared to the neat ABS sample, the obtained Young’s modulus values are reduced by 62.3 and 61.2% for F1 and F2 filler, respectively. The glassy and brittle structure of the microspheres renders the composites rigid and vulnerable to plastic deformation at lower stress. This observation is in line with other studies where expanded perlite was used as filler in polypropylene matrix [2,3]. It is noteworthy that, in all cases, the Young’s modulus value of the composite samples is marginally higher with the F2 filler. Increasing the infill ratio causes an increase in yield strength. Again, the F2 filler performs better compared to F1. This can be attributed to the fact that the F2 grade exhibits higher crushing resistance compared to F1. The neat ABS value of 41.86 MPa is exceeded by composite samples with F2 filler and infill ratios of 20 and 40 vol.% by 5.2 and 9.5%, respectively. This unusual increase in the yield strength with increasing infill ratio could be attributed to the presence of air voids and filler fragments, which was more intensive when infill ratio was between 5 and 20 vol.%, as revealed by the observation of the samples’ microstructure (Figure 4a–d).

#### 3.2.3. Thermal Properties

**Figure 7:**
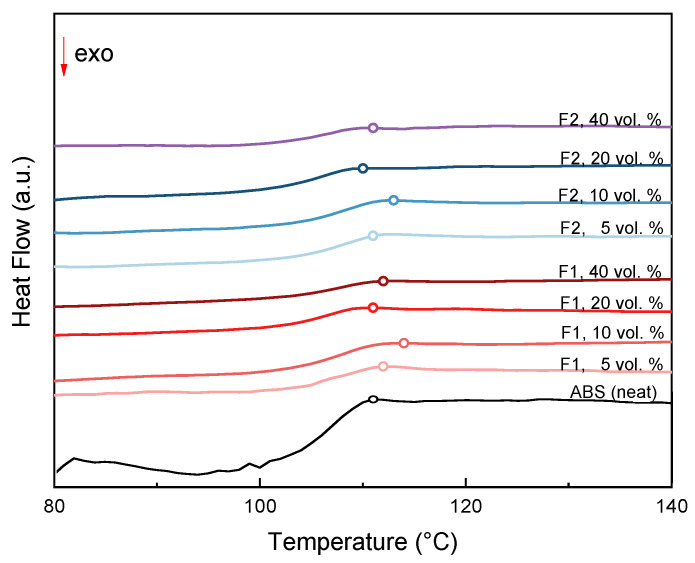
DSC diagrams for different ABS/filler composites and neat ABS. Open circles denote glass transition temperature.

The thermal behavior of the obtained composites and the reference ABS material was studied through Differential Scanning Calorimetry at a heating rate of 10 °K/min under a nitrogen atmosphere. The obtained diagrams are depicted in Figure 7 for composite samples with different ABS–filler compositions and for the reference ABS sample.

The glass transition near 110°C can be attributed to the polystyrene component of the ABS, and is considered the main characteristic of the material’s thermal behavior. Table 2 lists the glass transition temperatures as midpoints for all samples tested.

For both expanded perlite filler granulometries, as the proportion of the filler increases from 5 to 10% *v/v* there is a slight initial shift of the *T_g_* region to higher temperatures. The higher midpoint *T_g_* temperature for both granulometries is reached when the filler content is 10% *v/v*, then decreases to a temperature value near the midpoint *T_g_* temperature of the ABS base material.

### 3.3. Filament and FDM Samples

The studied properties of the filaments produced in a single screw extruder using neat ABS and various ABS–filler combinations are presented in this section. The filaments were subsequently used to construct dogbone samples appropriate for mechanical properties studies by employing the FDM 3D printing method. Corresponding results are presented in this section. In particular, −90 μm, 90–200 μm, and 200–350 μm granulometric grades of F3 type filler were used in proportions of 10 and 20 vol.% for the preparation of the composite filaments.

#### 3.3.1. Morphology

**Figure 8:**
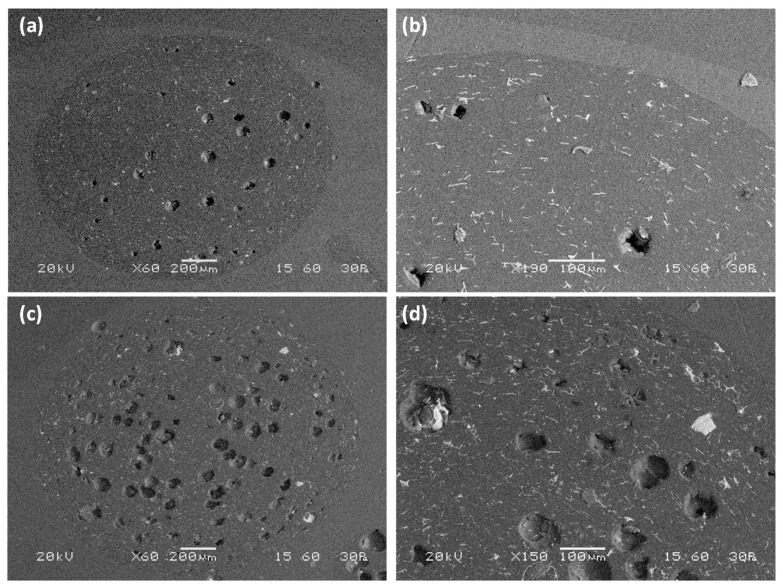
Morphology of two composite filament cross-sections produced using F3 filler: granulometry −90 μm and infill ratio of 10 vol.% on ×60 (**a**) and ×190 (**b**); granulometry of −90 μm and infill ratio of 20 vol.% on ×60 (**c**) and ×150 (**d**).

The morphology of two filament grades produced by incorporating F3 filler with −90 μm size and infill ratio of 10 vol.% and with 90–150 μm size and infill ratio of 20 vol.% are presented in Figure 8a,b and in Figure 8c,d, respectively.

For 10 vol.% infill ratio of −90 μm filler, a few pores can be identified in the filament cross-section (Figure 8a,b). The pore size ranges from a few microns to 90 μm, while a considerable number of microsphere fragments can be observed. The fragments appear as tiny white particles or white lines, as they are oriented parallel to the direction of filament extrusion and perpendicular to the filament cut. The spherical size of the pores and their size provide information about the presence of only a few filler particles. It appears that, for filler of a specific size, the majority of the microspheres did not survive the composite formulation procedure, leading to the production of a relatively compact composite consisting of only a few pores in the size of the filler and a sequence of thermoplastic material and filler fragments. More pores are identified for the filament that contains 20 vol.% filler (Figure 8c,d), thus indicating that more filler particles survived the filament preparation procedure. Again, a considerable number of fragments is present. Microsphere dispersion was satisfactory in both filaments,, indicating high homogeneity.

**Figure 9:**
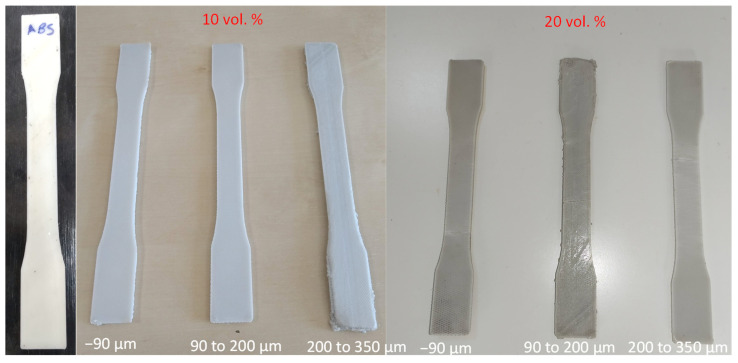
Dogbone samples printed using neat ABS and composite filament with different proportions of filler and different filler sizes.

Dogbone samples printed using composite filaments as well as filament that was produced solely with ABS beads are presented in Figure 9. All composite filaments possessed acceptable printability.

**Figure 10:**
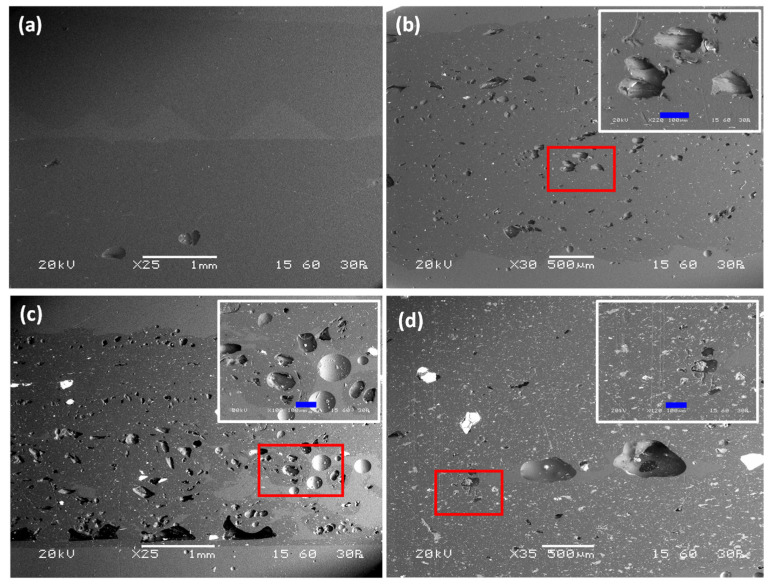
SEM of cross-sections of 3D printed dogbone samples constructed using: (**a**) neat ABS filament (×25), (**b**) composite filament with 10 vol.% of −90 μm microspheres (×30), (**c**) composite filament with 20 vol.% of 90–200 μm microspheres (×25), and (**d**) composite filament with 20 vol.% of 200–350 μm microspheres (×35). Detailed view of surface enclosed by the red rectangle is shown at the top left of (**b**–**d**) images. Length of blue bar is 100 μm.

Figure 10 depicts SEM images of cross-sections of the dogbone samples.

The sample made with pure ABS exhibits a compact microstructure with a lack of defects and air inclusions (Figure 10a). The use of composite filaments with −90 μm filler in a 10 vol.% infill ratio led to the appearance of voids homogenously distributed along the surface (Figure 10b). Although filler particles are not identified, the existence of voids should be attributed to the presence of filler because the size of the voids is comparable to that of the filler, which may possibly have been destroyed during polishing of the specimens. The dogbone sample containing filler with 90–200 μm diameter and 20 vol.% infill ratio presents a dense network of well-distributed voids with a size comparable to that of the filler (Figure 10c). Intact filler grains can be observed, together with tiny filler fragments distributed along the cross-section. Different morphology was obtained by the use of microspheres with 200–350 μm diameter and infill ratio of 20 vol.% (Figure 10d). The microstructure of the dogbone sample in the cross-section area presents a few voids with irregular shape which are is not consistent with either with the infill ratio or the size of the filler used. The presence of filler fragments is more intense compared to other samples, suggesting that the majority of the filler did not survive the extrusion procedure during the production of the composite filament, possibly due to its coarse size.

#### 3.3.2. Thermal Conductivity and Effusivity

**Figure 11:**
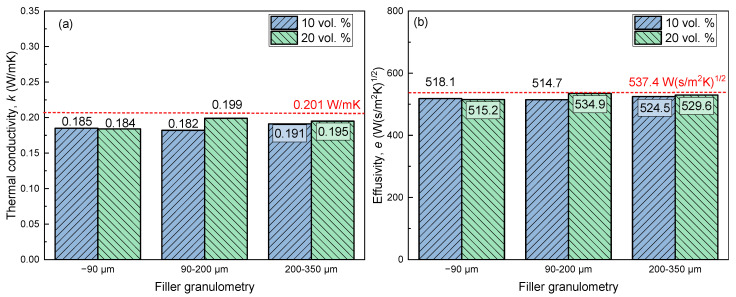
Thermal conductivity (**a**) and effusivity (**b**) of dogbone samples printed using composite filament (bars) and filament made of neat ABS (red dashed lines).

The thermal conductivity and effusivity of 10% and 20% *v/v* expanded ABS/perlite composite samples are depicted in Figure 11a,b for various filler granulometries.

As shown in Figure 11a, all samples printed exhibit slightly lower thermal conductivity compared to the pure ABS sample. This is in agreement with the morphological observation of the samples (Figure 10a–d), which shows that with respect to the incorporation of the filler, the use of composite filaments enhances the development of air voids, which are known to reduce the thermal conductivity of the samples. The sample printed using 90–200 μm filler grade with infill ratio of 10 vol.% possesses the minimum thermal conductivity value of 0.182 W/mK among all of the samples, which is almost 10% less than that of the neat ABS sample (0.201 W/mK). Similar to the thermal conductivity, the effusivity of the samples is reduced when the composite filament is used, with a minimum value of 514.7 W (s/m^2^K)^1/2^ observed when 90–200 μm filler is used with an infill ratio of 10 vol.%.

#### 3.3.3. Mechanical Properties of Printed Dogbone Samples

**Figure 12:**
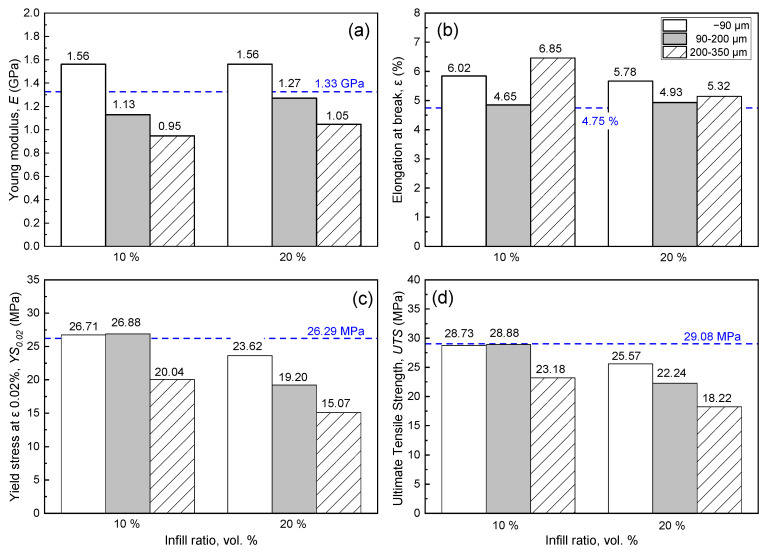
Mechanical properties of FDM dogbones printed using filament with different filler content and filler granulometry: (**a**) Young modulus, (**b**) elongation at break, (**c**) yield stress at elongation of 0.02%, and (**d**) ultimate tensile stress. Dashed horizontal lines depict relevant value for FDM dogbone made solely of ABS.

Figure 12 compares the Young’s modulus, elongation at break, yield stress at elongation of 0.02%, and ultimate tensile stress for dogbone samples printed using filaments produced by neat ABS and composite ones with different proportion of F3 filler and different filler size (−90 μm, 90–200 μm and 200–350 μm).

The use of the finest filler (<90 μm) allows for printing of dogbones with a higher Young’s modulus compared to the sample produced using neat ABS, resulting in an increase in the Young’s modulus by = 15.8–17.3% (Figure 12a). However, this was not the case when 90–200 μm and 200–350 μm fillers were used, which present lower Young’s modulus values compared to that of the neat ABS. The elongation of dogbones before breakage occurred is higher than the benchmark for 10 and 20 vol.% infill ratios (Figure 12b). The sample with coarse filler and minimum infill ratio presents the highest elongation at break (6.85%). Regardless of filler size, a systematic reduction in elongation at break is observed with increasing infill ratio. As was expected, the same trend is observed for the yield stress at elongation of 0.02% (Figure 12c). The use of composite filament with a filler size of 90–200 μm and 200–350 μm allows for printing of dogbone samples having practically the same yield stress at 0.02% elongation as the sample printed using neat ABS filament at a 10 vol.% infill ratio. A significant reduction of yield stress is identified for the coarser filler in all infill ratios, while for the maximum one (20 vol.%) the value is reduced by 42.7% (15.07 MPa) compared to the benchmark (26.29 MPa). Ultimate tensile stress constitutes another property that is adversely affected by the infill ratio, as revealed by the data presented in Figure 12d. The maximum ultimate tensile strength is observed for dogbone samples produced using filament with the minimum infill ratio (10 vol.%) and size <90 and 90–200 μm, while further increasing their content in filler causes systematic reduction of the ultimate tensile strength of the printed dogbone samples. Similar to the yield stress at 0.02% elongation, dogbone samples printed using filament with filler grade of −90 μm and 90–200 μm and 10 vol.% infill ratio obtained ultimate tensile stress that is practically the same as that of dogbone samples produced using neat ABS filament.

#### 3.3.4. Flammability Tests

**Figure 13:**
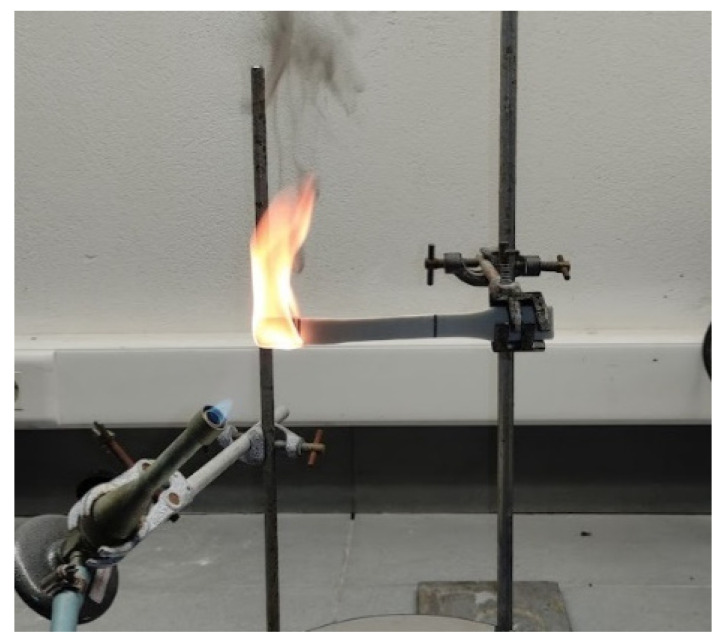
Snapshot of flammability testing of the sample with infill ratio of 10 vol.%.

The samples’ thicknesses, as well as the critical data obtained by subjecting three samples to the implemented flammability tests, are tabulated in Table 3. Moreover, a snapshot during experimental procedure is provided in Figure 13. For samples produced using composite filament, the filler diameter was <90 μm. All three samples fall into the HB category, presenting similar burning rates. Among all samples tested, the minimum linear burning rate was observed for the sample produced using composite filament with a 20 vol.% infill ratio.

## 4. Discussion and Conclusions

Mineral microspheres constitute a chemically inert, inorganic, and lightweight material with a foamy structure and spherical shape that acts multifunctionally when used as a filler in composites. The expansion process is fully controllable in a vertical electric furnace; together with control of the feed, this allows the production of fillers that are purpose-oriented in terms of size, side dispersion, and density. In this work, three grades of expanded perlite microspheres with different sizes and densities were produced and used as filler with ABS to form composites through compression molding and composite filaments for FDM in a screw extruder.

The incorporation of mineral microspheres in ABS matrix through the compression molding technique allows the production of composites with the filler well dispersed in the plastic matrix. As revealed by microstructure observation, the greater proportion of the incorporated filler survived the composites manufacturing technique, despite the considerable pressure exerted during sample preparation. This was verified by measurements of the composites’ density. The use of marginally heavier filler compared to the matrix plastic and increasing of the infill ratio led to the production of composites with progressively higher density, despite the fact that air voids were identified in certain samples. The mechanical properties of the produced composites were sensitive to the infill ratio as well as to the filler type. In all cases, the use of lighter filler led to the production of composite samples with lower compressive stiffness; however, the maximum stiffness of 0.74 GPa was observed for the sample with 40 vol.% infill ratio of F2 filler, which is 13% lower compared to that of the neat ABS sample (0.85 GPa). The same sample presented 9.5% higher yield strength compared to that of the sample produced with neat ABS (45.65 MPa and 41.67 MPa, respectively).

Regarding the formulation of composite filament for FDM using expanded microspheres, the filler size emerges as a critical parameter. Porous microspheres are considered a fragile material, and are vulnerable to breakage during compounding through screw extrusion, mainly due to shear between the molten material and the fixed surfaces and to the pressure exerted on the mixture in the compression zone of the extruder [21]. Of course, coarser grains are more likely to break while passing through the nozzle, producing a composite filament with considerable content of fragments, although it is known that perlite microspheres with low density possess limited compressive strength [12]. Indeed, the present study shows that among fillers with a size of <90 μm, 90–200 μm and 200–350 μm, the dogbones printed by the filament containing filler grade with the highest fineness and 20 vol.% infill ratio had the best mechanical properties. In this case, the Young’s modulus and elongation were higher compared to the benchmark (Young’s modulus of 1.56 and 1.33 GPa and elongation of 4.75% and 5.32%, for ABS and composite filament, respectively), while the ultimate tensile strength was practically the same. It is thus evident that for expanded microspheres the filler fineness is critical for the production of printed samples of acceptable quality, while it is possible that even higher infill ratios might be applicable in printed objects with mechanical properties at acceptable levels, provided that filler of an even higher fineness is used.

The environmental and economic aspects of the use of perlite microspheres in ABS composite are considerable. The embodied energy of ABS pellets is estimated at 95.30 MJ/kg, corresponding to 3.05 kg CO_2_ per kg, while for expanded perlite it is 10 MJ/kg and 0.52 kg CO_2_ per kg, respectively [22]. This great difference renders evident that the use of perlite as filler in the ABS matrix is beneficial to product sustainability. As to the economic aspects of the application, ABS is sold at USD 3000/m^3^. The price of expanded perlite is radically lower; conventionally expanded perlite with bulk density of 100–150 kg/m^3^ is sold a USD 100/m^3^. It has to be noted that due to the lower yield and the fine granulometry of the F1 and F2 grades compared to conventional perlite grades, the production price may reach USD 400/m^3^, which is again considerably lower compared to that of ABS, resulting in raw material cost savings. The positive impact of perlite in terms of material safety is shown by the fire rating tests, which revealed the marginal deceleration of burn rate in perlite–ABS composites.

## Figures and Tables

**Table 1:** Expansion temperature, density, and crushing resistance of three produced microsphere grades.

Sample	Expansion Temperature (°C)	Bulk Density (g∙cm^−3^)	Skeletal Density (g∙cm^−3^)	Crushing Resistance (MPa)
F1	990	0.375	1.24	4.7
F2	930	0.469	1.33	6.2
F3	1030	0.198	0.81	1.9

**Table 2:** The glass transition temperature (*T_g_*) for neat ABS and samples with different ABS–filler compositions.

Sample Composition	Glass Transition Temperature *T_g_* (°C)
Neat ABS	111.3
F1, 5 vol.%	112.3
F1, 10 vol.%	113.8
F1, 20 vol.%	110.6
F1, 40 vol.%	111.3
F2, 5 vol.%	110.3
F2, 10 vol.%	113.1
F2, 20 vol.%	110.2
F2, 40 vol.%	110.6

**Table 3:** Flammability of neat ABS sample and samples prepared with composite filaments of different filler content.

Sample Content in Filler, vol.%	Sample Thickness, mm	Linear Burning Rate *V*, mm/min	Flammability Rating
-	30	31.5	HB
10	32	33.4	HB
20	33	28.1	HB

## Data Availability

Not applicable.

## References

[1] Chatterjee K.K. (2013). Uses of Industrial Mineals, Rocks and Freshwater.

[2] Akin-Öktem G., Tanrisinibilir S., Tinçer T. (2001). Study on mechanical properties of perlite-filled gamma-irradiated polypropylene. J. Appl. Polym. Sci..

[3] Raji M., Nekhlaoui S., Hassani I.-E.E.A.E., Essassi E.M., Essabir H., Rodrigue D., Bouhfid R., Qaiss A.E.K. (2019). Utilization of volcanic amorphous aluminosilicate rocks (perlite) as alternative materials in lightweight composites. Compos. Part B Eng..

[4] Atagür M., Sarikanat M., Uysalman T., Polat O., Elbeyli İ.Y., Seki Y., Sever K. (2018). Mechanical, thermal, and viscoelastic investigations on expanded perlite–filled high-density polyethylene composite. J. Elastomers Plast..

[5] Lapčík L., Vašina M., Lapčíková B., Staněk M., Ovsík M., Murtaja Y. (2020). Study of the material engineering properties of high-density poly(ethylene)/perlite nanocomposite materials. Nanotechnol. Rev..

[6] De Oliveira A.G., Jandorno J.C., da Rocha E.B.D., de Sousa A.M.F., da Silva A.L.N. (2019). Evaluation of expanded perlite behavior in PS/Perlite composites. Appl. Clay Sci..

[7] Rallini M., Puri I., Torre L., Natali M. (2018). Thermal and ablation properties of EPDM based heat shielding materials modified with density reducer fillers. Compos. Part A Appl. Sci. Manuf..

[8] Alsaadi M., Erkliğ A. (2018). Effect of perlite particle contents on delamination toughness of S-glass fiber reinforced epoxy matrix composites. Compos. Part B Eng..

[9] Angelopoulos P.M., Samouhos M., Taxiarchou M. (2019). Functional fillers in composite filaments for fused filament fabrication: A review. Mater. Today Proc..

[10] Spoerk M., Sapkota J., Weingrill G., Fischinger T., Arbeiter F., Holzer C. (2017). Shrinkage and Warpage Optimization of Expanded-Perlite-Filled Polypropylene Composites in Extrusion-Based Additive Manufacturing. Macromol. Mater. Eng..

[11] Angelopoulos P.M., Kenanakis G., Viskadourakis Z., Tsakiridis P., Vasilopoulos K.C., Karakassides M.A., Taxiarchou M. (2021). Manufacturing of ABS/expanded perlite filament for 3D printing of lightweight components through fused deposition modeling. Mater. Today Proc..

[12] Angelopoulos P.M., Maliachova C., Papakonstantinou K., Taxiarchou M., Diplas S. (2016). Structural and physical characteristics of fine perlite expanded with a novel method in a vertical electric furnace. Miner. Process. Extr. Metall..

[13] Angelopoulos P.M., Gerogiorgis D.I., Paspaliaris I. (2013). Model-Based Sensitivity Analysis and Experimental Investigation of Perlite Grain Expansion in a Vertical Electrical Furnace. Ind. Eng. Chem. Res..

[14] Angelopoulos P.M., Taxiarchou M., Paspaliaris I. (2016). Production of durable expanded perlite microspheres in a Vertical Electrical Furnace. IOP Conf. Ser. Mater. Sci. Eng..

[15] (2002). Lightweight Aggregates, Part I: Lightweight Aggregates for Concrete, Mortar and Grout. Annex I: Determination of Crushing Resistance.

[16] Harris M., Potgieter J., Ray S., Archer R., Arif K.M. (2019). Acrylonitrile butadiene styrene and polypropylene blend with enhanced thermal and mechanical properties for fused filament fabrication. Materials.

[17] (2014). Standard Test Method for Tensile Properties of Plastics.

[18] (2003). Standard Test Method for Rate of Burning and/or Extent and Time of Burning of Plastics in a Horizontal Position.

[19] British Plastics Federation Acrylonitrile Butadiene Styrene (ABS) and Other Specialist Styrenics. https://www.bpf.co.uk/plastipedia/polymers/ABS_and_Other_Specialist_Styrenics.aspx.

[20] Aqzna S.S., Yeoh C.K., Idris M.S., Teh P.L., bin Hamzah K.A., Aw Y.Y., Atiqah T.N. (2018). Effect of different filler content of ABS–zinc ferrite composites on mechanical, electrical and thermal conductivity by using 3D printing. J. Vinyl Addit. Technol..

[21] Ariffin A., Ahmad M.S.B. (2011). Review: Single screw extruder in particulate filler composite. Polym. Plast. Technol. Eng..

[22] Hammond G., Jones C. (2011). The Inventory of Carbon and Energy (ICE): Embodied Energy and Carbon in Construction Materials (Ice). A BSRIA Guide.

